# Supplementary material for: Integrating Acupuncture into a Dialysis Center
Source: Healthcare (Basel). 2023 May 18;11(10):1463. doi: 10.3390/healthcare11101463 (PMC10217921; doi:10.3390/healthcare11101463)
Supplement: Supplementary file 1 [file healthcare-11-01463-s001.zip › healthcare-2343690-supplementary.pdf]

# Supplementary Materials Index

## Supplementary Document

Pre-and post- intervention survey 2

QUESTIONNAIRE CODE [ ]

As part of your participation in this study, please answer the following questions.

## A. PRE-INTERVENTION

1. **Do you know what acupuncture is?**
   YES [ ] NO [ ]

2. **Have you received any acupuncture treatment prior to your participation in the present study?**
   2.1 YES [ ] NO [ ]
   2.2 If so, how long ago has it been? < 3M [ ] > 3M [ ]
   2.3 And for what purpose? 2.3.1 Osteoarticular pain [ ] 2.3.2 Muscle soreness [ ] 2.3.3 Insomnia [ ]
   2.3.4 Anxiety [ ] 2.3.5 Generalized pain [ ] 2.3.6 Other [ ]

3. **Are you receptive to receiving an acupuncture treatment?**
   YES [ ] NO [ ]

## B. POST-INTERVENTION

**B1.** ***DEGREE OF DISCOMFORT***

***1. What is the degree of discomfort felt when puncturing the acupuncture needle?***

| 1 [ ] | 2 [ ] | 3 [ ] | 4 [ ] | 5 [ ] |
|---|---|---|---|---|
| Totally uncomfortable | Uncomfortable | Nor comfortable Nor Uncomfortable | Comfortable | Totally comfortable |

***2. What is the degree of discomfort during acupuncture treatment?***

| 1 [ ] | 2 [ ] | 3 [ ] | 4 [ ] | 5 [ ] |
|---|---|---|---|---|
| Totally uncomfortable | Uncomfortable | Nor comfortable Nor Uncomfortable | Comfortable | Totally comfortable |

***3. Throughout the acupuncture treatments, did you feel any adverse effects?***

YES [ ] NO [ ]

3.1 If yes, which one? ____________________________________________

***4. Would you recommend acupuncture treatment to others?***

YES [ ] NO [ ]

**B2.** ***INTERFERENCE WITH HEMODIALYSIS ROUTINE***

1. **In your opinion, have acupuncture treatments interfered with the hemodialysis routine?**

YES [ ] NO [ ]

1.1 If so, how did they interfere?

| 1 [ ] | 2 [ ] | 3 [ ] | 4 [ ] | 5 [ ] |
|---|---|---|---|---|
| Very negative | Negative | Neither negative nor positive | Positive | Very positive |

## QUESTIONNAIRE Healthcare Professionals CODE [ ]

1. **Age**
[ ]

2. **Gender**
Male [ ] Female [ ] Other [ ]

3. **Profession**
Doctor [ ] Nurse [ ]

What is the specialty? ________________________

4. **Education Level**
Bachelor´s degree [ ] Master's degree [ ] PhD [ ]

5. **Do you know what acupuncture is?**
YES [ ] NO [ ]

6. **Have you received any acupuncture treatment?**
6.1 YES [ ] NO [ ]
6.2 If so, how long ago? < 3M [ ] > 3M [ ]
6.3 And for what purpose? 6.3.1 Osteoarticular pain [ ] 6.3.2 Muscle soreness [ ] 6.3.3 Insomnia [ ]
6.3.4 Anxiety [ ] 6.3.5 Generalized pain [ ] 6.3.6 Other [ ]
6.4 Would you recommend acupuncture treatment to others? YES [ ] NO [ ]

7. **Would you be receptive to receiving an acupuncture treatment?**
YES [ ] NO [ ]

8. **What is your general opinion about acupuncture?**

| 1 [ ] | 2 [ ] | 3 [ ] | 4 [ ] | 5 [ ] |
|---|---|---|---|---|
| Very negative | Negative | Neither negative nor positive | Positive | Very positive |

9. **What is your opinion on the effectiveness and clinical applicability of acupuncture?**

| 1 [ ] | 2 [ ] | 3 [ ] | 4 [ ] | 5 [ ] |
|---|---|---|---|---|
| Very negative | Negative | Neither negative nor positive | Positive | Very positive |

10. **Have you ever suggested acupuncture to any patient?**
YES [ ] NO [ ]

11. **In your opinion, did the acupuncture treatments provided to patients interfere with the hemodialysis routine?**
YES [ ] NO [ ]
11.1 If so, how did they interfere?

| 1 [ ] | 2 [ ] | 3 [ ] | 4 [ ] | 5 [ ] |
|---|---|---|---|---|
| Very negative | Negative | Neither negative nor positive | Positive | Very positive |

11.2 Have you observed adverse events in patients resulting from acupuncture treatment?

YES [ ] NO [ ] 11.3 If so, which one?_______________

12. **What is your opinion on the integration of acupuncture in the care of patients on hemodialysis?**

| 1 [ ] | 2 [ ] | 3 [ ] | 4 [ ] | 5 [ ] |
|---|---|---|---|---|
| Very negative | Negative | Neither negative nor positive | Positive | Very positive |
